# Successful Intrasaccular Microcoil Embolization of a Lower Segment Pulmonary Artery Aneurysm in a Patient With Behcet’s Disease

**DOI:** 10.7759/cureus.62548

**Published:** 2024-06-17

**Authors:** Maroun Helou, Nael Al Irr

**Affiliations:** 1 General Practice, Faculty of Medicine and Health Science, An-Najah National University, Nablus, PSE; 2 Internal Medicine, Palestine Medical Complex, Ramallah, PSE

**Keywords:** internal medicine and rheumatology, pulmonary hemorrhage, aneurysm microcoiling, internal medicine, interventional radiology, behcet disease

## Abstract

Behcet's disease (BD) is an uncommon, long-term inflammatory condition characterized by recurring ulcers in the mouth and genital area, uveitis, and various systemic issues. One of the particularly rare but severe complications of this disease is the formation of pulmonary artery aneurysms (PAAs). Although these aneurysms are uncommon, they can lead to dangerous pulmonary hemorrhages (PHs), which are often fatal, requiring prompt diagnosis and intervention.

We present a case of lower segment PAA in an 18-year-old patient with recently diagnosed BD, presenting with life-threatening PH and managed successfully with microcoil embolization of the aneurysm and immunosuppressive (IS) medications, achieving stable remission without complications.

## Introduction

Aneurysms and pseudoaneurysms are rare abnormalities of the pulmonary arteries (PA). While their incidence is low, they represent potentially life-threatening conditions because their rupture can lead to massive hemoptysis and sudden death. They may present a challenge for prompt diagnosis and treatment [1]. Pulmonary artery aneurysms (PAA) are a rare complication in Behcet’s disease (BD) with around 1% prevalence, they are of particular concern because their rupture can lead to massive hemoptysis and sudden death [2].

This report details the case of an 18-year-old patient recently diagnosed with BD, who presented with a lower segment PAA complicated by pulmonary hemorrhage (PH). Given the complexities involved, a multidisciplinary approach was essential. The patient was successfully treated using microcoil embolization of the aneurysm combined with immunosuppressive (IS) therapy.

## Case presentation

An 18-year-old male presented to the emergency department with left leg pain and swelling that had developed over the course of a week. He further complained of large amount of fresh nasal bleeding, dizziness upon minimal exertion, generalized fatigue, coughing up streaks of blood and shortness of breath. On further inquiry, he revealed a recent history of recurrent painful oral ulcers treated with amoxicillin and local anesthetic, as well as recurrent genital ulcers managed with talc powder. Additionally, he had experienced two previous episodes of joint effusion in big toe and knee that resolved spontaneously, a progressive decline in vision necessitating glasses along with a history of 12 kg weight loss within one-year period. Despite the symptoms, the patient denied fever, headache, night sweats, chills, palpitations, chest pain, myalgia, urinary changes, vomiting and diarrhea.

Upon clinical examination, he appeared stable but was tachycardic with a heart rate of 145 beats per minute and normal blood pressure without orthostatic changes. His respiratory rate was within normal limits (18 breaths per minute), and oxygen saturation was satisfactory (97%). The patient had no signs of jugular venous distention, he had fine low pitch crepitations at right middle zone with good bilateral air entry. Physical examination revealed tenderness, warmth, and erythema in the left leg along with an appreciable size difference between his calves. His abdomen was soft, non-tender and non-distended.

Laboratory results upon presentation are demonstrated in Table 1. They indicated a drop of hemoglobin (Hgb) to 8 g/dl, and it was consistent with high erythrocyte sedimentation rate (ESR) microcytic hypochromic anemia. Arterial blood gas analysis (ABGs) was done and it showed mild respiratory alkalosis and the oxygen saturation was normal. Alveolar-arterial (A-a) gradient was expected to be 7 at his age, however it was calculated to be 48, which is high for his age, this indicates a ventilation-perfusion (V/Q) mismatch.

**Table 1:** Laboratory findings for the patient upon first presentation

Laboratory Parameter	Patient Lab Value	Reference Range
Hemoglobin (Hgb)	8	13.5-17 g/dl
Mean cell volume (MCV)	75	80-100 fL
Mean corpuscular hemoglobin (MCH)	23	27-32 pg
Erythrocyte sedimentation rate (ESR)	91	<15 mmhr
Activated partial thromboplastin time (aPTT)	85	25-35s
Prothrombin time (PT)	35	11-15s
International normalized ratio (INR)	2.6	-
Arterial blood gas analysis (ABGs)	pH: 7.45	pH: 7.35-7.45
	HCO_3_: 22	HCO_3_: 22-28
	PCO_2_: 27	PCO_2_: 35-45
	SaO_2_: 95%	SaO_2_ >95%

The findings have put vasculitis on top of differential diagnosis. Due to abnormalities in coagulation studies and fresh active nasal bleeding, it was decided to give the patient five units of fresh frozen plasma (FFP) initially; this improved his coagulations study findings.

Ultrasound (U/S) was done for limbs, it revealed dilated, decreased blood flow and decreased compressibility of superficial femoral vein (SFV) and popliteal vein, with normal blood flow in the common femoral vein (CFV), consistent with deep vein thrombosis (DVT). The diagnosis promoted to start the patient on subcutaneous enoxaparin sodium.

**Figure 1:**
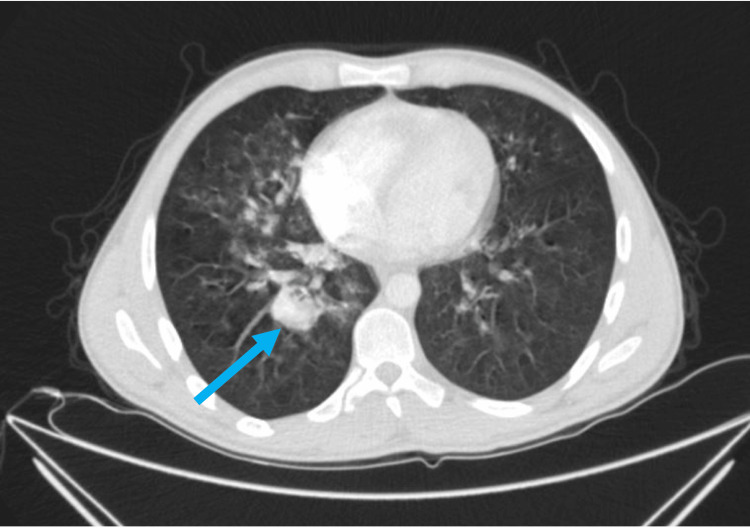
A chest CT scan with the blue arrow showing the pulmonary artery aneurysm (PAA) 2*3 cm

Further investigations, which included a chest CT angiography, were performed to rule out pulmonary embolism (PE) as a cause for hemoptysis, tachycardia, and tachypnea. The main pulmonary trunk and arteries were properly opacified without obvious filling defects, suggesting no evidence of PE. However, the segmental branches of both lower lobar arteries showed few aneurysms and wall thickening suggestive of vasculitis (Figure 1). Air space opacification seen bilaterally but predominantly in the right lung represented mostly an element of pulmonary hemorrhage (PH). Clinical, laboratory and imaging studies all aimed towards the diagnosis of BD diagnosis.

Rheumatologic laboratory investigations revealed positive lupus anticoagulant and positive HLA-B51 (HLA-B51 allele was tested by nested PCR technique in a DNA sample extracted from peripheral whole blood). Moreover, the patient had a negative rheumatoid factor RF, negative proteinase 3-antineutrophil cytoplasmic antibodies (PR3-ANCA) and myeloperoxidase (MPO)-ANCA, negative anti-dsDNA, anti SSA, anti Scl-70. Moreover, C3, C4 and immunoglobulins were all within the normal range. 

One day post admission, the patient developed rectal bleeding. Gastrointestinal (GI) endoscopy was performed which showed no active or recent GI bleed. Patient was clinically and vitally stable. He was discharged on aspirin, omeprazole, colchicine, azathioprine, prednisone and warfarin with instructions to keep the international normalized ratio (INR) between 2 and 3.

Despite initial improvement, the patient returned two weeks later to the emergency department with large amount of nasal bleeding and subsequent development of blood clots that were coughed up with fresh red blood. He was readmitted as a case of PH. Due to his presentation of PH and previous CT findings that suggested PAA, the internal medicine team determined that interventional radiology is necessary.

**Figure 2:**
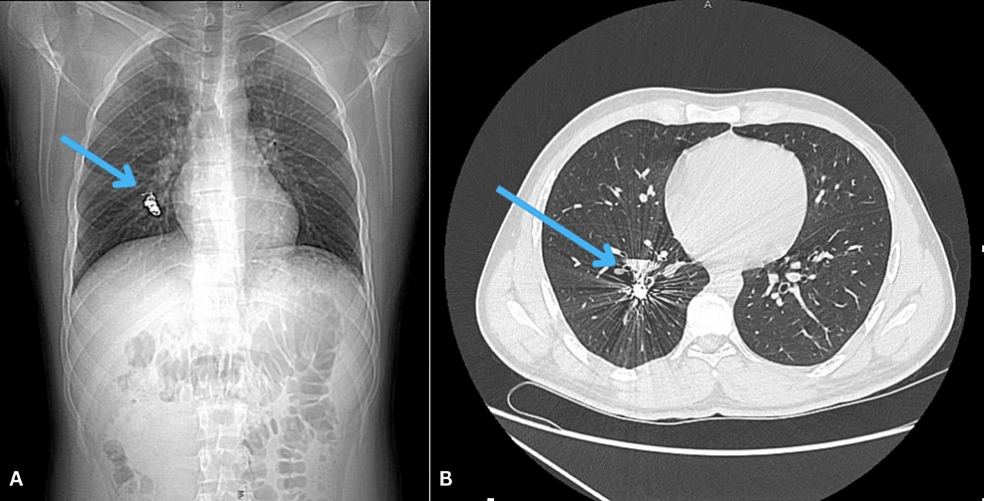
A) Post microcoil embolization. Pulmonary arteries (PA) chest and abdomen X-ray, with blue arrow showing the multiple micro coils in the aneurysm; B) Chest CT with arrow showing the micro coils

Angiography of the right PA showed an aneurysm size of 2x3 cm in the lower segment of the PA, and an embolization with intrasaccular insertion of multiple micro coils was performed. Figure 2 shows images of post microcoil embolization. Angiography of the left PA showed no aneurysms that require angioembolization. It was an uncomplicated procedure. Patient was then started on methylprednisolone, azithromycin, ceftriaxone, colchicine and omeprazole.

Despite the complexity of the case, the patient remained stable after radiological and medical intervention. A Long-term treatment plan was decided including azathioprine with infliximab. After multiple follow up appointments to the internal medicine clinic, patient sustained stability without further episodes of hemorrhages or ulcers.

## Discussion

The aforementioned 18-year-old patient initially presented to the emergency department exhibiting signs and symptoms of DVT in the left leg. Based on this presentation, he received treatment with anticoagulation therapy. Moreover, he was found to have high ESR microcytic hypochromic anemia, and subsequently developed massive hemoptysis leading to the diagnosis of PAAs through imaging studies. Positive laboratory findings coupled with clinical presentation supported the diagnosis of BD. Subsequently, the patient underwent medical management involving corticosteroids and infliximab. Interventional radiology performed aneurysm embolization using multiple micro coils, resulting in successful improvement and control of the patient’s condition.

BD is a chronic inflammatory condition affecting multiple bodily systems; its exact cause remains unknown. The demographic group at the highest risk for significant organ involvement is young males, particularly those under 30 years old [3]. It impacts arteries and veins of varying sizes, with venous complications being more frequently reported than arterial ones, which may reach up to 80% of the cases [4]. Involvement of the pulmonary vascular system can lead to severe consequences, it stands as a primary cause of mortality among patients with BD [2]. PAAs, characterized by localized dilation of these arteries, is a clear manifestation. While congenital heart disease is responsible for over half of the cases, other acquired causes vary widely and may include infections like tuberculosis and syphilis, pulmonary arterial hypertension, chronic PE, lung cancer, and certain medical procedures. BD can induce inflammation in the PA, leading to the formation of aneurysms [5].

In the present case, the patient’s progression of clinical picture was not consistent with any of the standard causes mentioned above. His case was consistent with an inflammatory vasculitic origin of the disease. Therefore, the BD diagnosis was based on recurrent oral ulcers, recurrent genital ulcers, DVT and pulmonary hemorrhage. Various diseases can lead to pulmonary arteritis. BD is known to affect both small and large arteries and veins. ANCA-associated vasculitides which encompass microscopic polyangiitis, granulomatosis with polyangiitis, and eosinophilic granulomatosis with polyangiitis, primarily impact small PA and can result in alveolar hemorrhage. Although CT findings in this case indicated hemorrhage from smaller PA, the likelihood of ANCA-associated vasculitides was deemed low due to the absence of other symptoms typically associated with the condition, such as fever, arthritis, airway or lung disease, kidney involvement and negative results for both MPO-ANCA and PR3-ANCA tests. Takayasu’s arteritis primarily affects larger blood vessels. However, it is more common in females where DVT is a rare complication. In instances where only the PA are affected without involvement of larger systemic arteries, it typically results in vascular stenosis, albeit rarely [6]. Our patient fulfilled the diagnostic criteria for BD.

Our subject patient initially commenced anticoagulation following the diagnosis of DVT, but this treatment was subsequently halted upon the onset of hemoptysis and nasal bleeding. There is insufficient controlled data or evidence to support the efficacy of anticoagulants, antiplatelet agents, or antifibrinolytic agents in managing DVT or arterial lesions associated with BD. In BD, venous thrombi adhere to vessel walls without causing emboli, and PE is rare despite frequent venous thrombosis. Therefore, the use of anticoagulants, antiplatelet agents, or antifibrinolytic agents is not recommended. Additionally, these agents should be avoided due to the potential risk of coexisting PAAs, which could lead to fatal bleeding. Previous research indicates that anticoagulants do not decrease the risk of recurrent venous thrombosis. Controlled trials are necessary to establish efficacy [7].

There is no conclusive evidence to direct the management of major vessel disease in BD. Immunosuppressants like infliximab and corticosteroids are primarirly used to manage PAAs. In a good number of patients, medical treatment alone leads to the resolution of the aneurysms. Tunaci et al. conducted a follow-up study using CT on 46 Behçet's patients with PA who received medical treatment. They discovered that 76% of the aneurysms were completely resolved within 3-42 months (with an average of 21 months) after treatment, while the remaining 24% just decreased in size [8].

Although medical treatment is effective, the prognosis and the mortality rates are influenced by the size of the aneurysm. According to Seyahi et al., patients with pulmonary aneurysms measuring ≥3 cm in diameter were at a higher risk of mortality due to complications compared to those with a diameter of <3 cm (p = 0.002) [9].

In cases where medical treatment fails or when patients present with life-threatening hemoptysis, endovascular management becomes a viable option for pulmonary aneurysms [10]. The subject patient presented with a lower segment PAA, which was successfully treated with coiling. Coil embolization, a minimally invasive procedure, was employed. Intra-saccular coil embolization preserves the pulmonary artery distal to the aneurysm, thus preserving lung function. However, there exists a risk of rupture, migration, or recurrence of hemoptysis [1]. Despite these potential complications, the patient has shown favorable progress one-year post-initial presentation.

While the potential hazards associated with surgical intervention for PAA are considerable, it can be deemed a vital measure in cases involving life-threatening hemoptysis. Ideally, surgical intervention ought to be deferred until the inflammatory process has abated; however, such circumstances are frequently impracticable, particularly in emergent situations like life-threatening hemoptysis. Given the elevated mortality rate observed in surgically managed cases, surgery should be considered only in instances where the patient's life is in imminent jeopardy [11].

## Conclusions

PAAs pose a significant risk of hemoptysis; they stand as the primary cause of mortality in BD. Nonetheless, a considerable portion of patients afflicted with PAAs exhibit positive prognoses following IS therapy. Corticosteroids and Infliximab stand out as the preferred initial treatments. For individuals who do not respond to medical intervention or those experiencing life-threatening hemoptysis, endovascular or surgical procedures become necessary. Endovascular approaches encompass embolization of the affected PA using coils. Generally, endovascular interventions are associated with fewer adverse effects compared to surgical alternatives.

## References

[1] Park HS, Chamarthy MR, Lamus D, Saboo SS, Sutphin PD, Kalva SP (2018). Pulmonary artery aneurysms: diagnosis & endovascular therapy. Cardiovasc Diagn Ther.

[2] Hamuryudan V, Yurdakul S, Moral F (1994). Pulmonary arterial aneurysms in Behçet's syndrome: a report of 24 cases. Br J Rheumatol.

[3] Kural-Seyahi E, Fresko I, Seyahi N (2003). The long-term mortality and morbidity of Behçet syndrome: a 2-decade outcome survey of 387 patients followed at a dedicated center. Medicine (Baltimore).

[4] Alibaz-Oner F, Direskeneli H (2019). Management of vascular Behçet's disease. Int J Rheum Dis.

[5] Kage H, Goto Y, Amano Y (2016). Development of pulmonary artery aneurysms due to Behçet's disease and resolution after treatment. Intern Med.

[6] Toledano K, Guralnik L, Lorber A (2011). Pulmonary arteries involvement in Takayasu's arteritis: two cases and literature review. Semin Arthritis Rheum.

[7] Hatemi G, Silman A, Bang D (2008). EULAR recommendations for the management of Behçet disease. Ann Rheum Dis.

[8] Tunaci M, Ozkorkmaz B, Tunaci A, Gül A, Engin G, Acunaş B (1999). CT findings of pulmonary artery aneurysms during treatment for Behçet's disease. AJR Am J Roentgenol.

[9] Seyahi E, Melikoglu M, Akman C (2012). Pulmonary artery involvement and associated lung disease in Behçet disease: a series of 47 patients. Medicine (Baltimore).

[10] Xie D, Chen C, Wang H, Xu Z, Jiang G (2015). Refractory pulmonary artery aneurysm in Behçet's disease. Ann Transl Med.

[11] Samreen I, Darji P, Genobaga S (2023). Pulmonary artery aneurysm in Behcet disease: medical, endovascular or surgical intervention. Cureus.

